# Impaired Function of HDAC6 Slows Down Axonal Growth and Interferes with Axon Initial Segment Development

**DOI:** 10.1371/journal.pone.0012908

**Published:** 2010-09-23

**Authors:** Mónica Tapia, Francisco Wandosell, Juan José Garrido

**Affiliations:** 1 Department of Molecular, Cellular and Developmental Neurobiology, Instituto Cajal, Consejo Superior de Investigaciones Científicas (CSIC), Madrid, Spain; 2 Centro de Biología Molecular “Severo Ochoa”, CSIC-UAM, Madrid, Spain; 3 Centro de Investigación Biomédica en Red sobre Enfermedades Neurodegenerativas (CIBERNED), Sevilla, Spain; CNRS UMR6543, Université de Nice, Sophia Antipolis, France

## Abstract

The development of morphological neuronal polarity starts by the formation and elongation of an axon. At the same time the axon initial segment (AIS) is generated and creates a diffusion barrier which differentiate axon and somatodendritic compartment. Different structural and functional proteins that contribute to the generation of neuronal action potential are concentrated at the axon initial segment. While axonal elongation is controlled by signalling pathways that regulate cytoskeleton through microtubule associated proteins and tubulin modifications, the microtubule cytoskeleton under the AIS is mostly unknown. Thus, understanding which proteins modify tubulin, where in the neuron and at which developmental stage is crucial to understanding how morphological and functional neuronal polarity is achieved. In this study performed in mice and using a well established model of murine cultured hippocampal neurons, we report that the tubulin deacetylase HDAC6 is localized at the distal region of the axon, and its inhibition with TSA or tubacin slows down axonal growth. Suppression of HDAC6 expression with HDAC6 shRNAs or expression of a non-active mutant of HDAC6 also reduces axonal length. Furthermore, HDAC6 inhibition or suppression avoids the concentration of ankyrinG and sodium channels at the axon initial segment (AIS). Moreover, treatment of mouse cultured hippocampal neurons with detergents to eliminate the soluble pool of microtubules identified a pool of detergent resistant acetylated microtubules at the AIS, not present at the rest of the axon. Inhibition or suppression of HDAC6 increases acetylation all along the axon and disrupts the specificity of AIS cytoskeleton, modifying the axonal distal gradient localization of KIF5C to a somatodendritic and axonal localization. In conclusion, our results reveal a new role of HDAC6 tubulin deacetylase as a regulator of microtubule characteristics in the axon distal region where axonal elongation takes place, and allowing the development of acetylated microtubules microdomains where HDAC6 is not concentrated, such as the axon initial segment.

## Introduction

The formation of the axon is the first step for a neuron to adopt its morphological and functional polarization. Axon growth and elongation depends, among other factors, on microtubules polymerization, which is controlled by microtubule associated proteins (MAPs), proteins that incorporate tubulin for polymerization, such as CRMP-2, and tubulin post-translational modifications (PTMs)[1]. While MAPs have been extensively studied, the role and participation of PTMs in axonal growth is largely unknown in neurons. Post-translational modifications of tubulin affect mainly the C-terminus of tubulin and include tyrosination, detyrosination, polyglutamylation, polyglycylation or Δ2 modification, [2], [3], [4]. The only PTM not related with the C-terminus is the acetylation of α-tubulin in lysine 40. The enzyme responsible for this acetylation has not been characterized, but it was recently proposed that Elp3, part of the elongator complex, may acetylate α-tubulin [5]. The levels of α-tubulin acetylation are higher in axon during its initiation [6] and during axonal elongation different tubulin PTMs show a differential localization pattern [7], [8]. Detyrosinated (Glu-tubulin) and/or acetylated tubulin are more resistant to depolymerizating agents and are the more stable microtubules [9]. Moreover, PTMs such as tubulin acetylation, alone or in combination with other PTMs, could have other specific functions not related to microtubule stability. For example, the molecular motor kinesin-1 binds preferentially to detyrosinated and acetylated microtubules [10], [11], [12], and two of the members of kinesin-1 family, KIF5C and KIF5B, have been identified at the axon during initial axon formation and at the axon initial segment [13], [14]. The axon initial segment (AIS) develops from the very beginning of axon formation [15], generates action potentials and filters axonal transport [16], [17], [18]. How this specialized domain is generated is poorly understood, however, treatment of neurons with taxol, which increases tubulin acetylation, can disrupt axonal polarized traffic [14]. Thus, together with the enrichment of acetylated tubulin at the axon, the axon cytoskeleton needs dynamic microtubules for its elongation and the specification of axonal domains and polarized cargo transport. This change in microtubules requires the participation of enzymes that reverse tubulin modifications, such as acetylation. Two enzymes that catalyze α-tubulin deacetylation have been characterized; HDAC6 and SIRT2 [19], [20]. Regarding nervous system, SIRT2 expression has been detected in neurite growth cones and in oligodendrocytes and plays a negative role in neurite growth in hippocampal neurons [21], [22]. On the other hand, HDAC6 expression has only been detected in neurons [22], is able to regulate dendritic growth [23], and its expression levels are reduced in mood disorder [24]. In non-neuronal cells, HDAC6 regulate microtubule growth velocity [25], localizes to the motile structures of polarized cells [26], is associated to the formation of cilia [27], participates in the regulation of actin structures [28] and deacetylates cortactin [28] and hsp90 [26]. In NIH 3T3 cells, HDAC6 controls the stability of the dynamic pool of microtubules, and HDAC6 inhibition with trichostatin A (TSA) delays their depolymerization [29], whereas HDAC6 overexpression increases the chemotactic motility [19]. Moreover, HDAC6 interacts with and control the phosphorylation of Tau [30], and it co-immunoprecipitates with the microtubule end-tracking protein EB1 [25].

The objective of our study was to determine the possible role of HDAC6 in axonal elongation and axon maturation, reflected in the establishment of the axon initial segment in cultured hippocampal neurons. We show that HDAC6 activity is necessary to maintain axonal growth rate and also for the polarized localization of proteins to the axon initial segment, which is enriched in detergent resistant acetylated tubulin microtubules. The lack of HDAC6 activity reduces axonal length, due to a lower axonal growth rate, and delocalizes ankyrinG and voltage gated sodium channels from the AIS. Our results suggest that the impaired concentration of axon initial segment proteins is due to the lost of AIS microtubules specificity when HDAC6 function is suppressed. These changes in microtubules are reflected by the alteration of KIF5C distribution along the neuron. Thus, HDAC6 play an important regulatory role in axonal elongation and also in the establishment of functional neuronal polarity.

## Materials and Methods

### Cell culture

Mice were obtained from the Centro de Biología Molecular and treated following the guidelines of Council of Europe Convention ETS123, recently revised as indicated in the Directive 86/609/EEC. In addition all protocols were approved by the institutional animal care and use committee. Hippocampal neurons were prepared as described previously [31]. Briefly, the hippocampi were obtained from E17 mouse embryos, washed in Ca^2+^/Mg^2+^-free HBSS, digested with 0.25% trypsin for 15 minutes at 37°C and dissociated. The cells were counted, diluted in MEM, 10% horse serum, 0.6% glucose, and plated on polylysine coated coverslips (1 mg/ml) at a density of 5,000 cells/cm^2^. Medium was changed after 2 hours for Neurobasal containing B-27 and glutamax-I and the neurons were cultured for 2 or more days. 5 µM 1-β-D-arabinofuranosylcytosine (AraC) was added after two days in culture. For biochemical experiments, hippocampal neurons were plated at a density of 200,000 cells/cm^2^, and cultured for 48 hours. Murine neuroblastoma N2a cells were originally obtained from the American Type Cell Culture (Neuro-2A, Reference: CCL131) and were grown at 37°C in 7% CO2, in DMEM supplemented with 10% foetal bovine serum (FBS, Invitrogen) and 2 mM glutamine.

### Reagents and Plasmids

The plasmids used for transient expression were: HDAC6-Flag (Addgene), HDAC6-GFP and HDAC6-H216A/H611A-GFP were obtained from Dr. Sanchez-Madrid [32], pEGFP-N1 (Clontech), shRNAs-HDAC6, and scramble shRNA in pGFP-V-RS vector (Origene). N6,2′-o-dibutyryladenosine 3′,5′-cyclic monophosphate sodium (dibutyryl-cAMP) were from Sigma. Tubacin and niltubacin were kindly provided by Dr. Ralph Mazitschek.

### Cell Transfection

N2a cells were transfected using Lipofectamine 2000 (Invitrogen) according to the manufacturer's instructions. For transfection, 500,000 cells were plated on a 60 mm-culture dish or on polylysine-coated coverslips (1 mg/ml). Co-transfections were performed with 9 µl Lipofectamine 2000 and 3 µg of total-DNA. After 5 hours, the medium was change to DMEM without FBS. N2a cells were induced to grow neurites by exposure to 1 mM dibutyryl-cAMP for 24 h in DMEM without FBS.

Primary hippocampal neurons were nucleofected using the Amaxa nucleofector kit for primary mammalian neural cells (Amaxa Bioscience) according to the manufacturer's instructions. Nucleofection was performed using 3 µg of total DNA. We used 3×10^6^ cells for each nucleofection and the cells were seeded at the density of 5×10^5^ cells/cm^2^. Nucleofected neurons were analyzed after 3 days in culture.

### Detergent extraction of hippocampal neurons

For detergent extractions, neurons were left in culture for 5 DIV and they were then treated with TSA. After 48 hours, the cells were washed briefly in phosphate buffer before extracting the cells with 0.5% Triton X-100 in cytoskeletal buffer (2 mM MgCl_2_, 10 mM EGTA, 60 mM Pipes pH 7.0) for 5 min at 37°C, as described previously [18]. After extraction the neurons were rinsed and fixed in PFA 4%. To analyze by Western-blot the acetylation levels of polymerized tubulin, neurons were rinsed once with sodium phosphate buffered saline (PBS) and once with PHEM buffer (60 mM PIPES, 25 mM HEPES, 10 mM EGTA, 2 mM MgCl_2_ [pH 6.9]) and they were then extracted for 5 min with PHEM buffer containing 10 µM taxol, protease inhibitors, 0.1% dimethyl sulfoxide and 0.2% Triton X-100 [33]. Extracted cells were then lysed and homogenized in a buffer containing 20 mM HEPES [pH 7.4], 100 mM NaCl, 100 mM NaF, 1% Triton X-100, 1 mM sodium orthovanadate, 10 mM EDTA and Complete inhibitor protease cocktail (Roche Diagnostics).

### Immunocytochemistry

Neurons were fixed in 4% paraformaldehyde for 20 minutes and washed in PBS. For immunodetection, the coverslips were treated with 50 mM NH_4_Cl and incubated in blocking buffer (0.22% gelatin, 0.1% Triton X-100 in PBS) to avoid non-specific binding. The cells were then incubated for 1 hour at room temperature with the primary antibodies diluted in blocking buffer. The primary antibodies used were: mouse anti-acetylated-α-tubulin (1∶2000), mouse anti-tyrosinated-α-tubulin (1∶2000), mouse anti-MAP2 (1∶400) and mouse anti-PanNaCh (1∶75) from Sigma; mouse anti-tau-1 (1∶1000); and rabbit anti-MAP2 (1∶500) from Chemicon; mouse anti-ankyrinG (1∶100) from Invitrogen; rabbit anti-HDAC6 (1∶400) and rabbit anti-KIF5C (1∶200) from Abcam. The secondary antibodies used were a donkey anti-mouse or anti-rabbit Alexa-Fluor-488, 594 or 647 (1∶500). F-actin was stained using Alexa-Fluor-594-conjugated Phalloidin (1∶100). After staining, the coverslips were mounted with Fluoromount-G (Southern-Biotech) and the images were acquired on a vertical Axioskop-2plus microscope (Zeiss) or a confocal microscope (LSM510, Zeiss) under the same conditions to compare intensities. Differential interference contrast (DIC) images were obtained in a LSM510 confocal microscope. Analysis of axon and dendrite length and fluorescence intensities was performed with the Neuron J and ImageJ software tools. Images were prepared for presentation using the Adobe CS3 software.

### Western blot analysis

Protein samples were prepared from high density hippocampal neuron cultures or murine neuroblastoma N2a. The cells were lysed and homogenized in a buffer containing 20 mM HEPES [pH 7.4], 100 mM NaCl, 100 mM NaF, 1% Triton X-100, 1 mM sodium orthovanadate, 10 mM EDTA and Complete inhibitor protease cocktail (Roche Diagnostics). The proteins were then separated on 8% SDS-PAGE gels and transferred to nitrocellulose membranes. The membranes were incubated overnight at 4°C with primary antibodies in blocking solution (PBS, 0.2% Tween, 5% non-fat milk or 10% FBS). The antibodies used to probe the membranes were: mouse anti-α-tubulin (1∶10.000), mouse anti-acetylated-α-tubulin (1∶10.000), mouse anti-β-actin (1∶10.000), rabbit anti-Flag M2 (1∶200, Sigma), mouse anti-PanNaCh (1∶200, Sigma) and rabbit anti-HDAC6 (1∶200, Santa Cruz Biotechnology) or rabbit anti-HDAC6 (1∶200, Abcam). Secondary antibodies were from Amersham. Antibody binding was then visualized by ECL (Amersham) and densitometry was performed with an imaging densitometer (GS-800, Bio-Rad).

### Statistical Analysis

All experiments were repeated at least three times and the results are presented as the mean and standard error of the mean (S.E.M). The number of neurons analyzed in each set of data is indicated in figure legends. Statistical differences between experimental conditions were analyzed by a t-test or paired t-test using the Sigmaplot 9.0 software.

## Results

### Tubulin deacetylases inhibition reduces axonal elongation

**Figure 1:**
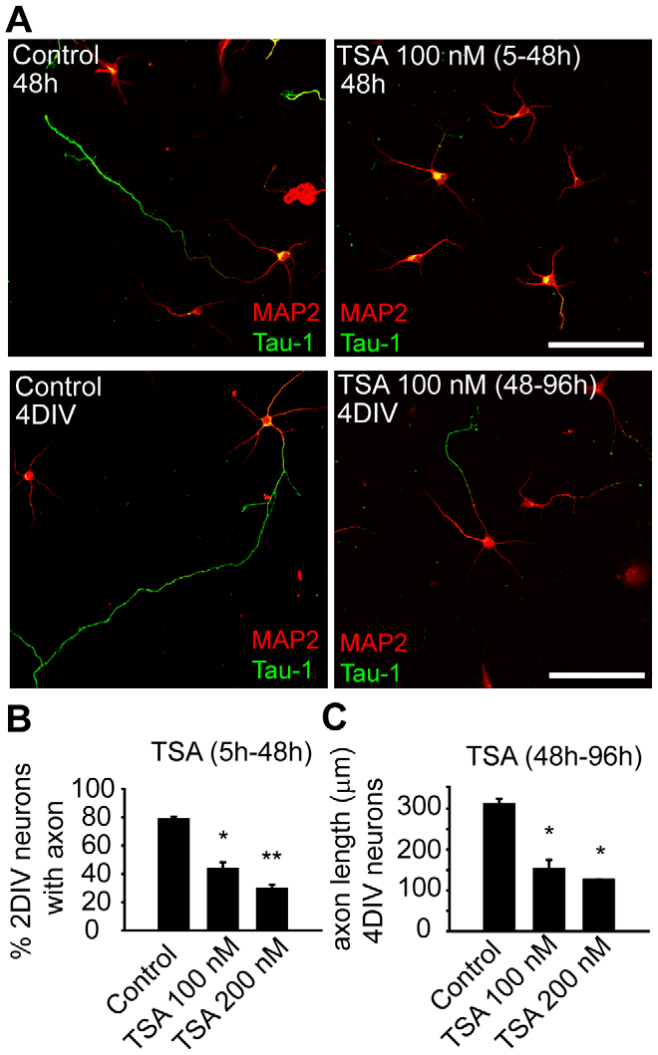
Inhibition of Tubulin deacetylases impairs axon elongation. (A) 2 DIV hippocampal neurons cultured for 48 hours in the presence or absence of TSA (100 nM), and 4 DIV hippocampal neurons treated with TSA from 48 to 96 hours. Neurons were stained with antibodies against MAP2 and Tau-1 to define the somatodendritic and axonal compartments. Scale bar  = 50 µm. (B) Percentage of 2 DIV neurons that develop an axon, when treated with TSA (100 or 200 nM) from 5 hours to 48 hours. Data represent the mean ± SEM of 3 independent experiments (300 neurons/experimental condition and experiment). *p<0.05, **p<0.01, paired t-test. (C) Mean axon length of 4 DIV hippocampal neurons treated with TSA (100 or 200 nM) from 48 to 96 hours. Data represent the mean ± SEM of 3 independent experiments (100 neurons/experimental condition and experiment). *p<0.05, paired t-test.

To assess whether tubulin deacetylases play a role in axonal growth, cultured hippocampal neurons were treated with trichostatin A (TSA), which inhibit HDACs but not sirtuins. Treatment of neurons with 100 or 200 nM TSA for 2 DIV increased tubulin acetylation (Figure S1). Control neurons showed an acetylated tubulin staining along the axon with an increasing distal gradient, while TSA treatment disrupted this polarization and increased tubulin acetylation all along the neuron, both in the axon and in the somatodendritic domain (Figure S1). Thus, we assessed whether tubulin acetylation due to HDACs inhibition was affecting axon initial specification or only axonal growth. TSA was added to cultured hippocampal neurons 5 hours after plating, before axon is specified, at a concentration of 100 or 200 nM and kept for 48 hours (Figure 1A). As shown in figure 1B, only 43.44±4.76% or 29.28±2.95% of the neurons treated with 100 or 200 nM TSA, respectively, were able to develop an axon (a tau-1 positive process) when compared to control neurons (78.37±2.08%). Next, 2 DIV neurons (that have developed an axon) were exposed to TSA until 4 DIV (Figure 1A). While control neurons developed axons of 310.57±13.48 µm, the length of the axon developed by neurons treated with 100 nM or 200 nM TSA was 151.85±22.85 µm and 126.01±0.29 µm, respectively (Figure 1C).

**Figure 2:**
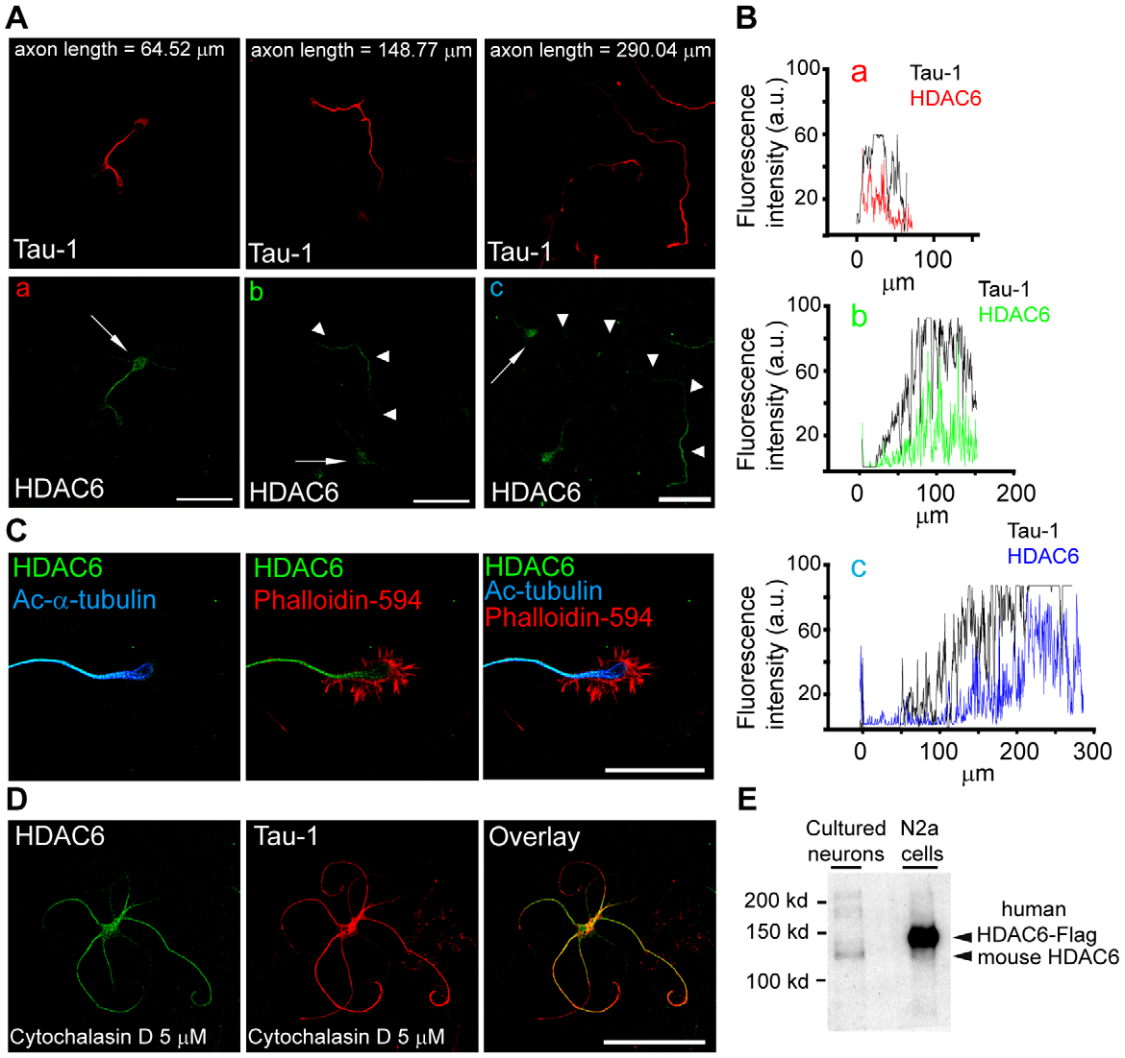
HDAC6 localizes to axons and it is distributed in the distal region of axons during elongation. (A) Axonal localization of HDAC6 (green) during axon elongation, where the axon is identified as the tau-1 (red) positive process. Arrows indicate neuronal soma position and arrow heads the axon. Scale bar = 50 µm. (B) Fluorescence intensity along the axons of neurons (a, b and c) shown in A. HDAC6 intensity is shown in colour lines and their respective tau-1 intensities in black lines. (C) Distal region of the axon and growth cone of a 1.5 DIV hippocampal neuron stained for HDAC6 (green), F-actin (red) and acetylated-α-tubulin (blue). Scale bar  = 50 µm. (D) 3 DIV neuron treated with cytochalasin D from 1 to 3 DIV and stained for Tau-1 and HDAC6. (E) Western-blot of 3 DIV cultured hippocampal neurons and human HDAC6-Flag transfected N2a cells. Mouse and human HDAC6 were detected using the same antibody that the one used for immunocytochemistry (panel A). Note the difference in molecular weight between human and mouse HDAC6.

Then we analyzed the expression of HDAC6, the only HDAC specifically related with tubulin deacetylation. The antibody used for immunocytochemistry was first analyzed for its HDAC6 specificity in culture hippocampal neurons extracts or in extracts of N2a cells expressing human HDAC6-Flag (Figure 2E). The antibody specifically detects both the mouse and the human HDAC6 proteins, and colocalizes with HDAC6-GFP expression in neurons (Figure S3). When we analyzed the expression of HDAC6 during axon elongation, HDAC6 was detected in the incipient axon of 1.5 DIV neurons, while its expression was mainly located to the distal axon in 3 and 4 DIV neurons, co-localizing with tau-1 in neurons with a longer axon (Figure 2A and B). Also, in a model of neurons with multiple axons, such as cytochalasin D treatment, HDAC6 was detected in all processes following the same pattern that tau-1 (Figure 2D). Even more, HDAC6 expression was co-localized with acetylated-α-tubulin, its putative substrate (Figure 2C).

### HDAC6 inhibition or suppression delays axon formation and decreases elongation

**Figure 3:**
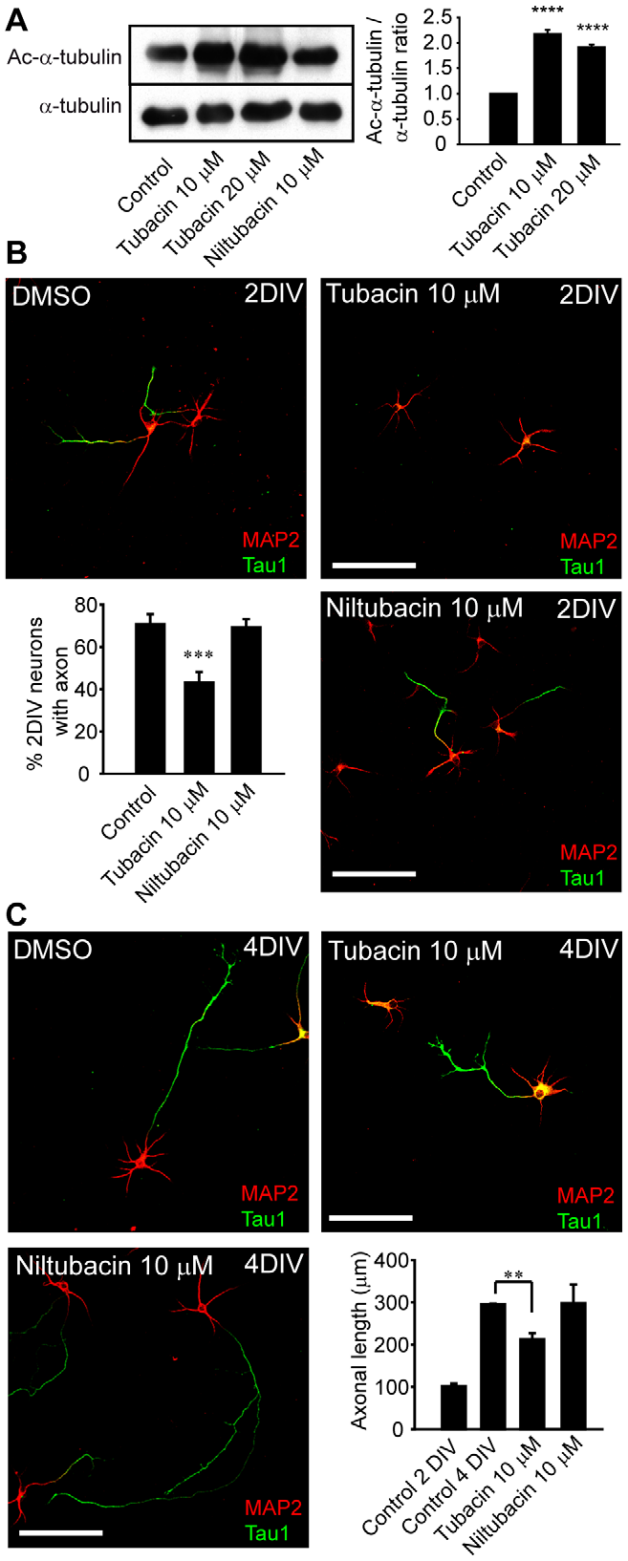
HDAC6 activity inhibition reduces the rate of axon elongation. (A) α-tubulin acetylation in 2 DIV hippocampal neurons treated with the HDAC6 specific inhibitor tubacin, its non-active analog, niltubacin, or its vehicle, DMSO (control). The graph represents the mean ± SEM of the acetylated-α-tubulin/α-tubulin ratio normalized to control in 3 independent experiments. ****p<0.0001, paired t-test. (B) 2 DIV hippocampal neurons cultured in the presence of vehicle (DMSO), the HDAC6 inhibitor (tubacin, 10 µM) and its inactive compound (niltubacin, 10 µM). Neurons were stained for MAP2 and tau-1. Scale bar  = 100 µm. The graph represents the percentage of neurons with a tau-1 positive process. Data represent the mean ± SEM of 3 independent experiments (500 neurons/experimental condition and experiment). ***p<0.001, paired t-test. (C) 4 DIV hippocampal neurons cultured from 48 hours to 4 days in vitro in the presence of DMSO, tubacin (10 µM) or niltubacin (10 µM), and stained as indicated in (B). Scale bar  = 100 µm. The graph represents the axonal length of control neurons fixed at 2 DIV or 4 DIV, and of 4 DIV neurons treated from 2 DIV to 4 DIV with tubacin or niltubacin. Data represent the mean ± SEM of 3 independent experiments (150 neurons/experimental condition and experiment). **p<0.01, paired t-test.

Then, we studied whether the effects of TSA in axonal growth could be due to HDAC6 inhibition. To inhibit HDAC6 activity we used tubacin, a highly specific inhibitor of HDAC6 that does not affect the level of histone acetylation or patterns of gene-expression [34]. As a control, we used neurons treated with DMSO, the vehicle, or with niltubacin, an inactive derivate of tubacin [34]. Tubacin treatment clearly inhibited HDAC6, increasing the levels of acetylated tubulin, while niltubacin did not affect tubulin acetylation (Figure 3A and Figure S1). To assess the effect of HDAC6 inhibition on axonal growth, low density cultures of neurons were exposed to tubacin from 5 hours after plating to 2 DIV (Figure 3B), or from 2 DIV after plating to 4 DIV (Figure 3C). Our results show that a significant percentage of neurons treated from 5 hours in vitro with 10 µM tubacin (43.08±5.05%) were unable to elongate an axon (a tau-1 positive process), while 70.75±4.77% of control neurons developed an axon (Figure 3B). By contrast, exposing neurons to niltubacin (10 µM) did not affect axon growth, which was similar to that in control neurons (69.17±3.95%, Figure 3B). To determine whether inhibiting HDAC6 stopped or slowed down axon growth, tubacin or niltubacin were added to neurons that had previously developed an axon. After 48 hours in vitro, the mean length of the axon in control neurons was 102.15±5.86 µ µm (Figure 3C) and after 2 additional days in vitro, their axons had reached a length of 295.45±1.47 µm. When maintained from 2 DIV to 4 DIV in the presence of 10 µM tubacin, the mean length of the axon developed by the neurons fell to 212.45±14.6 µm. Treatment with 10 µM niltubacin did not affect the rate of axon growth that had a mean length of 297.79±44.49 µm (Figure 3C).

**Figure 4:**
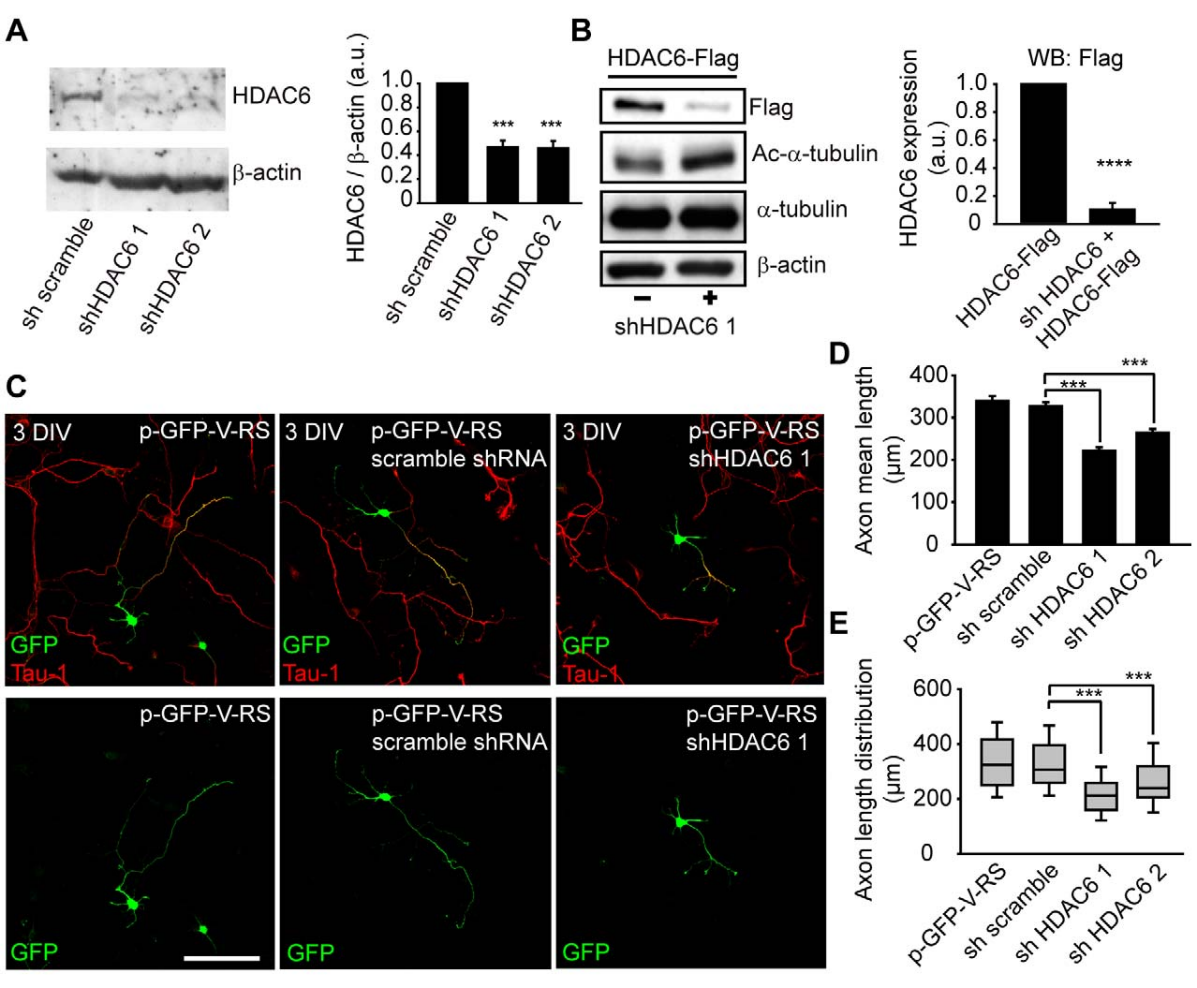
Suppression of HDAC6 by interference shRNAs reduces axonal elongation in hippocampal neurons. (A) Endogenous HDAC6 in N2a cells is suppressed by the expression of two different HDAC6 shRNAs. Graph represents the mean and SEM of HDAC6 expression levels normalized to β-actin levels in 3 different experiments. ***p<0.001, paired t-test. (B) HDAC6 shRNA suppresses the expression of exogenous HDAC6-Flag in N2a cells and increases acetylated-α-tubulin expression. Graph represents the reduction in HDAC6 expression in N2a cells measured with the anti-Flag antibody. Data represent the mean and SEM of 3 independent experiments. ****p<0.0001, ***p<0.001, paired t-test. (C) 3 DIV hippocampal neurons nucleofected with GFP plasmids expressing HDAC6 interference RNAs or scramble shRNA. Neurons were stained with anti-Tau-1 antibody to identify the axon. Scale bar  = 100 µm. (D) Quantification of the mean axonal length of 3 DIV hippocampal neurons nucleofected with the GFP plasmids expressing GFP, scramble shRNA, HDAC6 shRNA 1 or HDAC6 shRNA 2. (E) Box-plot shows the axonal length distribution of neurons quantified in D. ***p<0.001, paired t-test.

To fully confirm our pharmacological experiments we analyzed axonal growth and elongation in the absence of HDAC6. Due to the relative low percentage of neurons that can be nucleofected, we first checked that two different interference shRNAs against HDAC6 can reduce endogenous HDAC6 expression in Neuro-2a cells (Figure 4A), and that a HDAC6 shRNA can reduce exogenous HDAC6-Flag expression in Neuro-2a cells (Figure 4B). HDAC6 shRNAs reduced endogenous HDAC6 expression approximately a 50% (Figure 4A), and also the expression of exogenous HDAC6-Flag (Figure 4B), increasing the levels of acetylated-α-tubulin in Neuro-2a cells and neurons (Figure 4B and Figure S1).

Next, we nucleofected hippocampal neurons with plasmids expressing two shRNAs against HDAC6, a scramble shRNA and GFP, in order to confirme the role of HDAC6 in axonal growth. After 3 days, we stained neurons with the tau-1 antibody (Figure 4C) and we quantified axonal length (Figure 4D and 4E). HDAC6 expression was reduced in neurons nucleofected with shRNA HDAC6 and absent from axons (Figure S2). Neurons nucleofected with plasmids expressing GFP or scramble shRNA had a mean ± s.e.m. axonal length of 339.25±11.81 µm or 326.90±9.61 µm, respectively. In the case, of neurons nucleofected with HDAC6 shRNA 1 or HDAC6 shRNA 2, axonal length was significantly reduced to 221.33±8.11 µm and 263.99±9.21 µm, respectively (Figure 4C, 4D and 4E). In parallel to shRNA experiments, we nucleofected neurons with HDAC6-GFP or a non-active form of HDAC6 (HDAC6-H216A/H611A-GFP), mutated in both deacetylase domains. HDAC6-GFP location did mimic that of endogenous HDAC6 (Figure S3) and was localized in axon with a distal gradient, as previously shown for endogenous HDAC6. However, the non-active HDAC6-GFP was homogenously distributed along the axon and HDAC6 gradient along the axon was lost (Figure S3). HDAC6-GFP expression did not change axonal length compared to GFP expressing neurons, while the expression of the mutated HDAC6-GFP reduced axonal length in the same proportion as tubacin or HDAC6 shRNAs (Figure S3).

Moreover, we tested the effect of interfering with HDAC6 in a model of dibutyryl-cAMP (db-cAMP) induced neuritogenesis in N2a cells (Figure S4). N2a cells expressing HDAC6 shRNA showed a significant lower percentage of differentiation (neurite extension) in the presence of 1 mM db-cAMP (25.06±4.57%), compared to N2a cells transfected with the GFP control plasmid (36.13±1.99%) (Figure S4). In parallel, we measured the percentage of N2a cells differentiating when expressing HDAC6-GFP or the non-active HDAC6-GFP mutant (Figure S4). Confirming the results obtained with the HDAC6 shRNA constructs, there was a significative reduction in the percentage of N2a cells differentiating in the presence of db-cAMP when expressing the non-active HDAC6-GFP mutant (35.65±7.85%) compared to control N2a cells expressing HDAC6-GFP (57.7±10.4%).

### Inhibition or suppression of HDAC6 impairs the concentration of voltage gated sodium channels and ankyrinG at the axon initial segment

In view of the results indicating a role of HDAC6 in axonal elongation, we checked whether a later step in axonal maturation, the development of the axon initial segment was affected by changes in microtubule characteristics.

**Figure 5:**
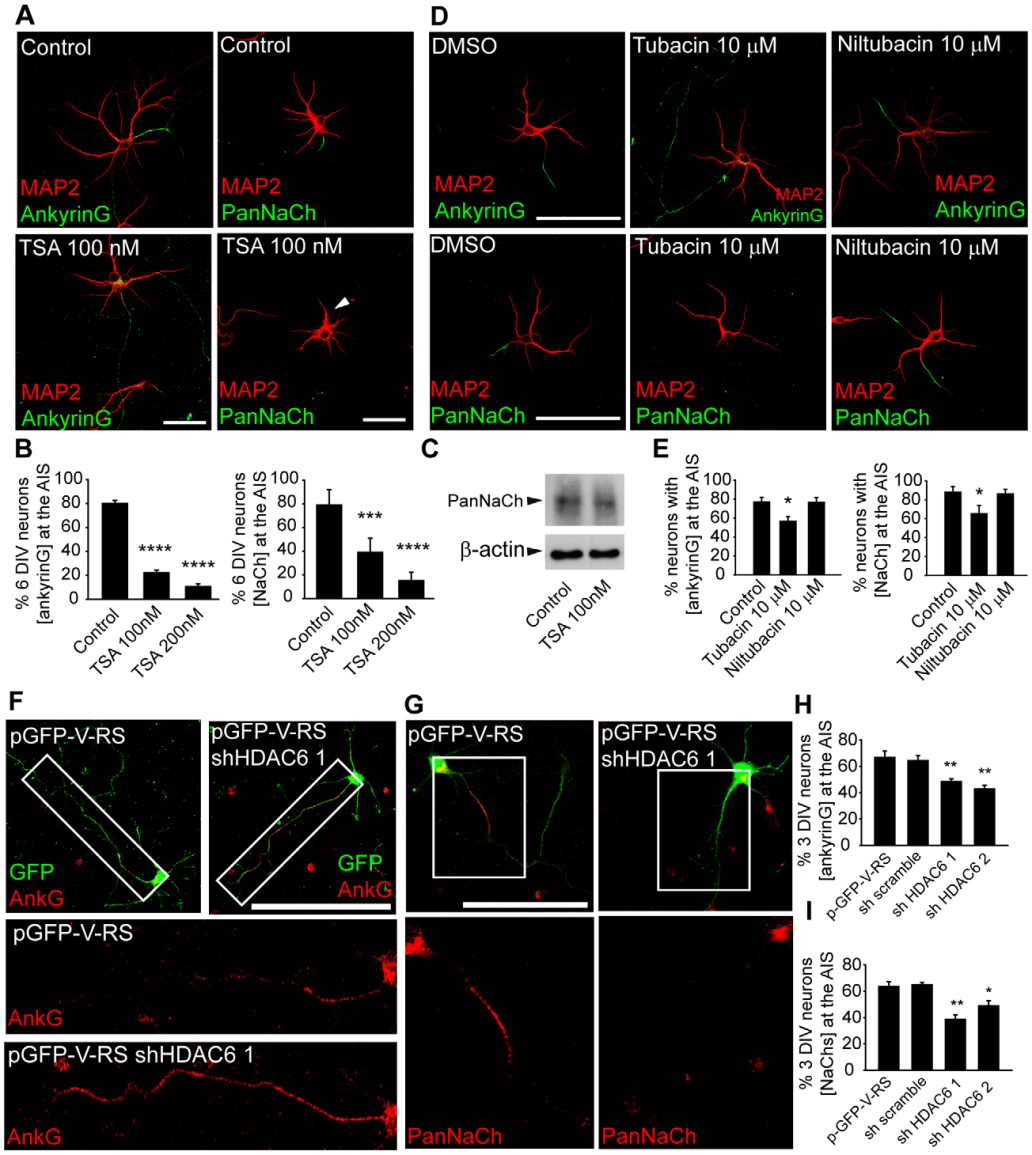
HDAC6 inhibition or suppression interfere the concentration of ankyrinG and voltage dependent sodium channels at the axon initial segment. (A) Control or TSA treated 6 DIV hippocampal neurons stained with the somatodendritic marker, MAP2 (red), and the AIS markers, ankyrinG or PanNaCh (green). Control neurons concentrate ankyrinG and sodium channels in the AIS, while in TSA treated neurons ankyrinG staining is distributed all along the axon, and voltage gated sodium channels staining is very low or lost. Arrow head indicate the position of the axon. Scale bar  = 50 µm. (B) Percentage of neurons with ankyrinG or voltage gated sodium channels concentrated in the AIS. Data represent the mean ± SEM of 3 independent experiments (500 neurons/experimental condition and experiment). ***p<0.001, ****p<0.0001, paired t-test. (C) Western-blot shows the expression levels of the α-subunit of voltage gated sodium channels in control and 100 nM TSA treated neurons compared to β-actin expression levels. (D) 6 DIV hippocampal neurons treated with DMSO, tubacin or niltubacin from 3 DIV to 6 DIV, and then stained for MAP2 (red) and ankyrinG or sodium channels (green). (E) Percentage of neurons that show a localized concentration of ankyrinG or sodium channels at the axon initial segment. The graphs represent the mean ± SEM of 3 independent experiments (600 neurons/experimental condition and experiment). *p<0.05, paired t-test. Scale bar = 100 µm. (F) 3 DIV hippocampal neurons nucleofected with an HDAC6 shRNA plasmid or the control plasmid. After 3 days in culture, neurons were stained for ankyrinG and the neuronal morphology was distinguished by GFP fluorescence. Rectangles indicate the magnified zone shown in lower panels. Lower panels show a magnification of both neurons along the axon with ankyrinG concentrated at the initial region of the axon (control neurons, pGFP-V-RS) or along the axon (pGFP-V-RS-shRNA-HDAC6 nucleofected neurons). Scale bar  = 100 µm. (G) 3 DIV hippocampal neurons nucleofected as in E and stained with an anti-PanNaCh antibody. Square inserts show a magnification of both neurons at the level of the AIS. Scale bar = 100 µm. (H, I) Percentage of GFP, scramble shRNA or HDAC6 shRNAs nucleofected neurons that concentrate ankyrinG (G) or sodium channels (H) in the AIS after 3 days in culture. Data are the mean ± SEM of 3 independent experiments (100 neurons/experimental condition and experiment). * p<0.05, **p<0.01, t-test.

Then, we studied whether tubulin deacetylases, and particularly HDAC6, could affect the localization of important proteins for AIS constitution and function, such as, ankyrinG and voltage gated sodium channels. The AIS starts to form from the beginning of axon growth [15] and it can be considered to be mature as a filter for cytoplasmic traffic at 5 DIV [17]. Thus, cultured hippocampal neurons were exposed to 100 or 200 nM TSA from 3 DIV to 6 DIV. Neurons treated with TSA showed a loss of ankyrinG and sodium channels concentration at the AIS (Figure 5A and 5B). Indeed, TSA reduced the proportion of neurons in which ankyrinG concentrated at the AIS from 80.01±2.73% in control neurons, to 22.19±2.27% and 10.58±2.32%, in the presence of 100 or 200 nM TSA, respectively (Figure 5B). Moreover, ankyrinG distribution was detected along the axon (Figure 5A). Sodium channels, which are concentrated at the AIS by ankyrinG interaction, were only concentrated at the AIS in 39±12% (100 nM TSA) or 15±7% (200 nM TSA) of these neurons when compared to the control neurons (79±13%, Figure 5A and 5B). As sodium channels expression levels are not affected (Figure 5C), the absence of sodium channel staining, in TSA treated neurons, can be explained by the fact that sodium channels are diffuse and under the limit of PanNaCh antibody detection limits.

Next, we studied whether this effect was due to HDAC6 inhibition. First, we treated 3 DIV hippocampal neurons with 10 µM tubacin until 6 DIV. Control neurons showed concentrated voltage gated sodium channels and ankyrinG at the AIS in 88.16±5.76% and 76.99±4.93%, respectively (Figure 5D and 5E). Exposure to 10 µM tubacin reduced the number of neurons in which sodium channels concentrated at the AIS to 65.19±8.89% (Figure 5D and 5E). The concentration of AnkyrinG at the AIS was also affected and only 56.47±4.94% of neurons treated with 10 µM tubacin concentrated ankyrinG in this domain. AnkyrinG staining was also diffuse along the axon, as above shown in TSA treated neurons. Niltubacin (10 µM) treatment did not change ankyrinG or sodium channels distribution (Figure 5D and 5E).

Finally, we analyzed AIS proteins distribution after suppression of HDAC6 expression. As previously described, neurons were nucleofected with scramble or HDAC6 shRNAs, as well as, the control plasmid. Neurons were cultured for 3 days and the percentage of neurons that concentrated sodium channels and ankyrinG at the AIS was analyzed (Figure 5F-I). Only 48.41±2.2% and 42.72±2.99% of HDAC6 interference RNA 1 or 2 nucleofected neurons showed a concentration of ankyrinG at the AIS. In the same experiments, only 38.66±3.47% and 48.83±4.02% of neurons nucleofected with HDAC6 interference RNA 1 or 2 had a concentration of sodium channels at the AIS. Control or scramble shRNA nucleofected neurons showed higher percentage of neurons with concentrated ankyrinG at the AIS (66.72±4.87, in control neurons, and % 64.29±3.87% in shRNA scramble neurons) and sodium channels (63.62±3.78, in control neurons, and % 65.01±1.69%, in shRNA scramble neurons). The reduced concentration of ankyrinG due to HDAC6 interference expression was associated with an ankyrinG staining along the axon (Figure 5F), similar to that produce by tubacin or TSA treatment.

### Tubulin deacetylases participate in maintaining specific characteristics of axon initial segment microtubules

**Figure 6:**
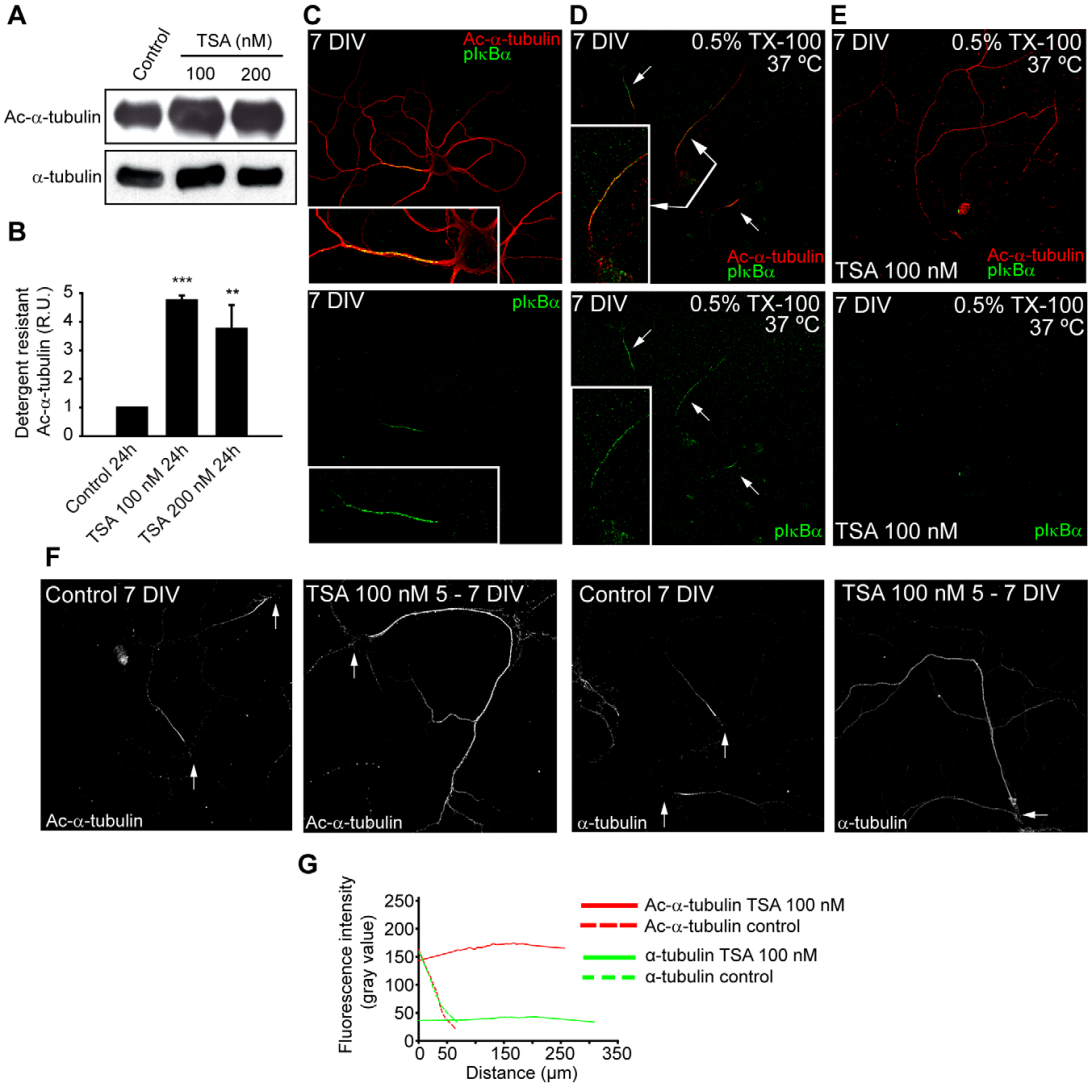
Tubulin deacetylase inhibition disrupts the axon initial segment specific enrichment in detergent resistant acetylated microtubules. (A) Acetylated-α-tubulin levels in microtubules resistant to PHEM buffer extraction in control and TSA treated neurons. (B) Graph represents the mean ± SEM of α-tubulin acetylation levels from 3 experiments as indicated in A. **p<0.01, *** p<0.001, t-test. (C) Acetylated-α-tubulin expression in 7 DIV hippocampal neurons. Axon initial segment is stained with the pIκBα antibody. (D) 7 DIV hippocampal neurons fixed after extraction with 0.5% Triton X-100 in cytoskeletal buffer (2 mM MgCl2, 10 mM EGTA, 60 mM Pipes pH 7.0) for 5 min at 37°C. Arrows indicate the axon initial segments, stained with acetylated-α-tubulin (red) and pIκBα (green). Box shows an amplification of the indicated axon initial segment. (E) Acetylated-α-tubulin localization in 100 nM TSA treated neurons extracted as indicated in D. Note that acetylated tubulin is not further restricted to the AIS and located along the axon. (F) Acetylated-α-tubulin and total α-tubulin immunostaining of control or 100 nM TSA treated neurons extracted with 0.5% Triton X-100. Arrows indicate the position of neuronal somas. (G) Graph represents the fluorescence intensity of acetylated-α-tubulin (red lines) and α-tubulin (green lines) along the stained axon in control (dotted lines) and TSA (straight lines) treated neurons shown in F. Intensity lines are the result of smoothing the data obtained with the ImageJ program using the Sigmaplot software.

HDAC6 inhibition or suppression impairs the tethering of AIS proteins; nevertheless HDAC6 is not localized at the axon initial segment in any analyzed stage from 1 to 15 DIV (Figure 2A and unpublished data). In order to understand the mechanism by which HDAC6 inhibition or suppression affects axon initial segment proteins concentration at the AIS, we analyzed whether the HDAC6 presence at the distal region of the axon and its absence from the axon initial segment could confer these microtubules different characteristics compared to microtubules at the axon initial segment. Thus, we analyzed whether AIS microtubules characteristics were different from the rest of the axon. AIS contained proteins are characterized by their resistance to detergent extraction [15], [16], [18]. In fixed 7 DIV neurons (Figure 6C), acetylated tubulin was distributed throughout the neuron, but when neurons were treated with non-ionic detergent (TX-100) prior to fixation, acetylated tubulin was essentially restricted to the AIS and co-localized with a marker of the axon initial segment, pIκBα (Figure 6D, 6F and 6G). This restriction of acetylated microtubules to the AIS was eliminated when 7 DIV hippocampal neurons were treated with 100 nM TSA during 48 hours prior to extraction, and acetylated-α-tubulin staining was homogeneously distributed all along the axons (Figure 6E–G). At the same time, pIκBα staining was not concentrated and under detection limits of the antibody (Figure 6E). We confirmed by western-blot that TSA had significantly increased the levels of acetylated-α-tubulin in the non-extracted resistant microtubules (Figure 6A and 6B).

### The lack of HDAC6 activity alters the KIF5C overall distribution along the neuron

**Figure 7:**
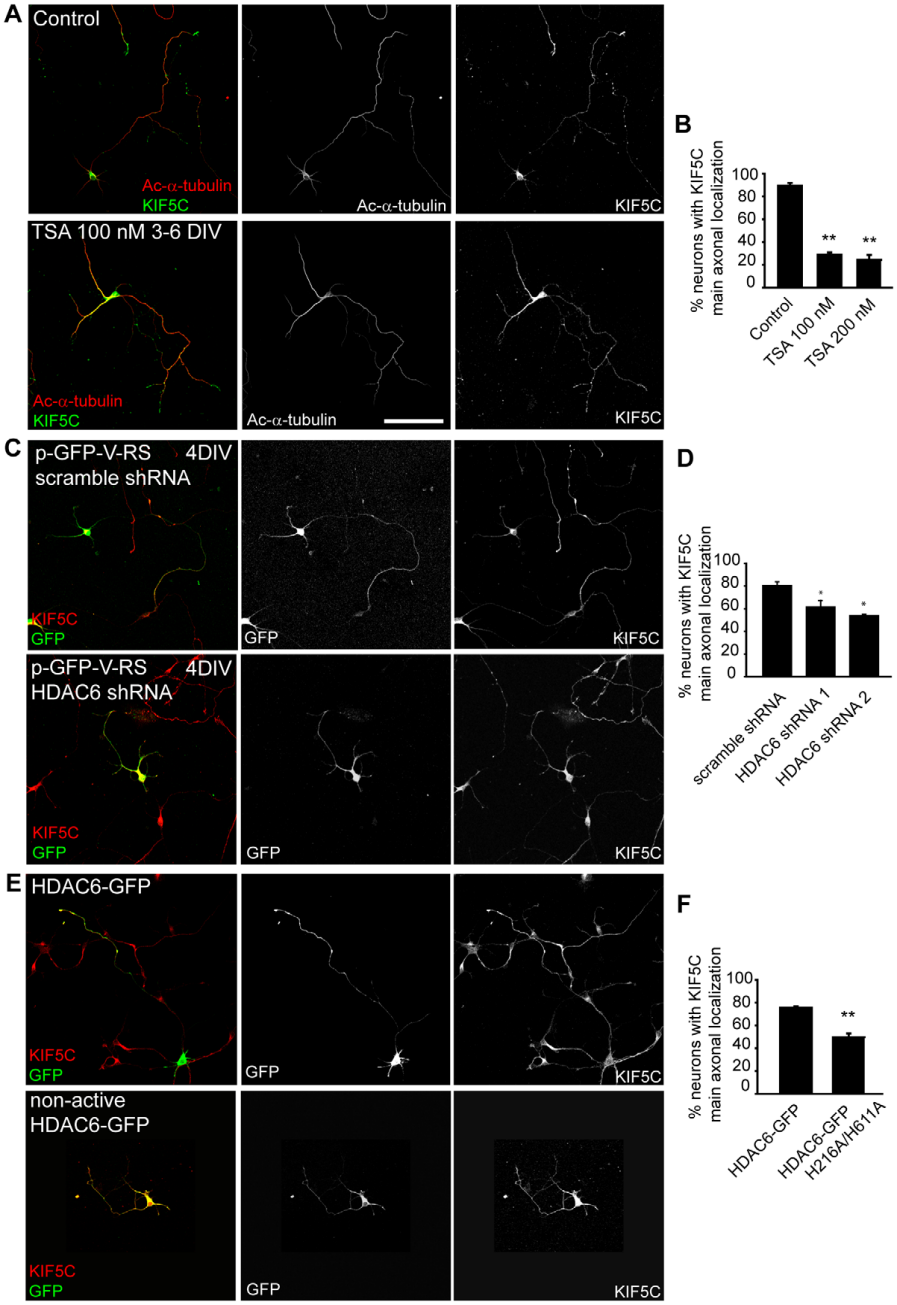
The absence of HDAC6 activity alters the distribution of KIF5C along the neuron. (A) KIF5C localization and acetylated-α-tubulin staining in 6 DIV hippocampal neurons cultured in the absence of TSA, or treated with 100 nM TSA from 3 to 6 DIV. Scale bar  = 100 µm. (B) Percentage of neurons with a KIF5C with a main axonal localization as shown in A (control neuron). **p<0.01 (t-test). (C) KIF5C localization in 4 DIV hippocampal neurons nucleofected before plating with HDAC6 interference shRNA or scramble. GFP fluorescence indicates the nucleofected neurons. (D) Percentage of scramble or HDAC6 interference shRNA nucleofected neurons with a KIF5C with a main axonal localization. *p<0.05 (t-test). (E) KIF5C localization in 3 DIV HDAC6-GFP or HDAC6 non-active mutant nucleofected neurons. (F) Percentage of neurons showing a KIF5C with a main axonal localization in neurons expressing HDAC6-GFP or an HDAC6-GFP non-active mutant. **p<0.01 (t-test). All data represent the mean ± SEM of three experiments and at least 100 neurons by condition and experiment.

Acetylated tubulin is also related to protein transport and serves as an interaction point for kinesins. Kinesin-1 has been related with axonal growth, axon initial segment, and with acetylated tubulin. Thus, we checked whether microtubules modifications due to the absence of HDAC6 could affect the location of the molecular motor, KIF5C. First, we treated 3 DIV hippocampal neurons with 100 nM TSA and analyzed the distribution of KIF5C at 6 DIV. While KIF5C staining was mainly detected in the distal region of the axon in 89.53±2.28% of control neurons, only 29.00±2.07% of TSA treated neurons presented a clear axonal distal gradient of KIF5C (Figure 7A and 7B). The modification of KIF5C axonal gradient was characterized by a homogeneous staining along the axon and a somato-dendritic staining of KIF5C, associated to an increased tubulin acetylation in both domains (Figure 7A and 7B). However, TSA treatment did not impede KIF5C localization at axonal growth cones (Figure 7A). Concomitantly, after HDAC6 suppression KIF5C axonal gradient in 4 DIV neurons was only observed in around a 55% of neurons compared to scramble shRNA nucleofected neurons (Figure 7C and D). Regarding HDAC6 function, KIF5C was polarized to the axon in 75.53±1.07% of HDAC6-GFP nucleofected neurons, while only 49.7±3.29% of non-active HDAC6-GFP presented an axonal polarization of KIF5C (Figure 7E and 7F). Both, after suppression of HDAC6 or the expression of the non-active HDAC6, KIF5C was also observed at axonal tips, clearly augmented in the somatodendritic domain and homogeneously distributed along the axon.

These results suggest that the reduction in axonal growth due to HDAC6 suppression can be related, in some degree, to the altered distribution of motor proteins, after modification of microtubules domains along the axon.

## Discussion

HDAC6 has been involved in multiple regulatory events related to development or disease, interacting with different proteins. Thus, it is possible that HDAC6 could have different functions in different developmental stages and different physiological or pathological conditions. While HDAC6 function during brain development has not been extensively studied, HDAC6 has an important role in the regulation of mechanisms related with different neurodegenerative diseases in adult brain. HDAC6 ubiquitin binding activity is necessary for the recruitment of autophagic material to aggresomes and its degradation by proteasome [35]. HDAC6 is necessary for the degradation of aggregated hungtingtin [36], regulates the traffic of parkin, a Parkinson linked protein [37], and also HDAC6 mRNA levels are significantly decreased in bipolar disorder patients [24].

We have focussed our study in the role of HDAC6 during the first stages of axonal growth in hippocampal neurons. Our data show a role for HDAC6 during the initial development of axons, where HDAC6 is necessary for axonal elongation and the development of the axonal initial segment. In this developmental context and based in our observations, we believe that HDAC6 localization at the distal region of the axon regulate microtubules characteristics in this region, at least through its deacetylase domains. Our data show that the absence of HDAC6 activity in hippocampal neurons, due to pharmacological inhibition or to the genetic targeted elimination of HDAC6, slows down axonal growth and generates an overall increased tubulin acetylation. It has been previously reported in non-neuronal cells that HDAC6 inhibition can reduce microtubule growth velocity [25]. The authors propose that this reduction is independent of microtubules acetylation generated by HDAC6 inhibition, and may be the result of the generation of a “leaky cap” at the plus-end of microtubules, impeding both attachment and detachment of tubulin subunits [25]. From our data we can conclude that HDAC6 activity is necessary for axonal growth, however we can not exclude that in neurons the increased acetylation due to HDAC6 inhibition can play an important role in neuronal development. One important difference between non-neuronal cells and neurons is the expression of Tau, an important regulator of HDAC6 [38]. In fact, our results show that HDAC6 colocalizes in neurons with acetylated microtubules and with Tau. A recent study has pointed that the ratio between acetylated tubulin versus tyrosinated tubulin is higher in the neurite specified as axon [6]. Thus, an increment of acetylated tubulin levels in all the neurites after HDAC6 inhibition could explain the lack of axonal growth due to the lost of polarized distribution of acetylated versus tyrosinated tubulin ratio at the neurite previously specified as axon, and the subsequent absence of polarized axonal traffic. Our data show that the sustained inhibition of HDAC6 or its suppression during axonal growth, increases microtubules acetylation and modifies the overall distribution of at least a motor protein, KIF5C, which is one of the first proteins to be present in the axon growth cone during initial neuronal polarization [13] and has also been found to co-localize with microtubules at the axon initial segment [14]. Our data are supported by the fact that treatment of neurons with taxol, that increases tubulin acetylation, generates a non polarized distribution of kinesin-1 [14], [39]. The same study shows that TSA or tubacin treatment for short times (around 3 hours) did not modify exogenous kinesin-1 localization [39]. However, we show that a sustained inhibition of HDAC6 with TSA or tubacin (during at least 48 hours) did altere the distribution of endogenous KIF5C along the neuron without impairing KIF5C arrival to growth cones. In fact, a rise in microtubule acetylation due to HDAC6 inhibition increases the binding of kinesin-1 and their transport speed [12] and generates a non-polarized distribution of JIP, an interacting protein of kinesin-1. Two recent studies have shown that kinesin-1 family members, KIF5B or KIF5C, bind and navigate preferentially on detyrosinated tubulin and on acetylated microtubules [10], [11].

HDAC6 has different functional domains, two deacetylase domains and an ubiquitin binding domain. Impaired function of HDAC6 also reduces dendrites length by a mechanism involving ubiquitin binding domain. HDAC6 binds Cdc20 and is necessary for the polyubiquitination of Cdc20, which stimulates the activity of centrosomal Cdc20-APC complex promoting the degradation of Id1, and drives the differentiation of dendrites [23]. The Cdc20-APC complex has not been localized in axons and is located at the centrosome, structure that is not necessary for axonal elongation [40]. It is possible that HDAC6 can regulate axon growth and dendrite growth forming different complexes in different subcellular locations. Our observations demonstrate a role of the HDAC6 deacetylase activity in the regulation of axon development, and inhibition of HDAC6 deacetylase or HDAC6 suppression has a similar effect in axonal growth reduction. Moreover, we have shown that HDAC6 co-localizes with the Tau-1 marker at the axon, and HDAC6 interaction with Tau can change Tau phosphorylation [30]. Tau is a neural specific protein not expressed in other non-neuronal cells, what may explain differences observed between neuronal and non-neuronal cells studies of HDAC6. The co-localization of HDAC6 and Tau-1 in the more dynamic domain of the axon suggest a role of both proteins in promoting the microtubules cytoskeleton characteristics necessary for axonal growth and transport. In fact, Tau can inhibit HDACs activity and is necessary for the binding of HDAC6 to microtubules [38]. Tau also binds to the dynactin complex and this interaction promotes the attachment of the complex to microtubules [41]. Interestingly, HDAC6 also interacts with the dynactin complex [35], and so it can regulate protein transport to the axon. The phosphatase PP1 has also been found in a complex with HDAC6 [42]. PP1 is recruited to microtubules by Tau, dephosphorylates Tau in other epitopes and also other MAPs and plays a role in the regulation of microtubules [43]. Thus, it is possible that HDAC6 activity may be regulated by MAPs, or can regulate the interaction of MAPs with microtubules. In this sense, an important regulator of MAPs, GSK3, interacts and activates HDAC6 deacetylase activity [44]. Interestingly, GSK3 inhibition decreases HDAC6 activity [44], but also is able to impair axon formation [45].

Under pathological conditions, HDAC6 may play a function in neuronal regeneration under restrictive conditions for axonal growth, and it has been proposed that HDAC6 inhibition can partially neutralize the inhibitory effects of myelin associated glycoprotein (MAG) in the initial growth of cortical neurons neurites [46]. However, our data show that HDAC6 activity is necessary for the initial development of the axon in hippocampal neurons in conditions where glial cells are not present or their role is reduced, as it happens in early development. One plausible explanation is that, in conditions where MAG is activating receptors and signaling proteins, HDAC6 plays another role coupling the MAG signaling to microtubules. HDAC6 inhibition could then abolish the MAG negative effect in neurites growth. In fact, it has been described in podosomes that increased Rho activity can significantly increase HDAC6 deacetylase activity [47].

Regarding the involvement of HDAC6 in the regulation of the axon initial segment (AIS), a highly important structure for the axonal identity and neuronal function [16], [48], we show that the AIS microtubules resistant to detergent extraction are highly acetylated. Previous studies have shown that microtubules in different axonal regions have different post-translational modifications [8], [49]. This specificity of AIS microtubules is disrupted by HDAC6 inhibition, which increases acetylation of microtubules all along the axon and confers the property of detergent extraction resistance to these microtubules. Thus, proteins that are allocated to the AIS, such as ankyrinG, change their distribution along the axon after HDAC6 inhibition or suppression, allowing other ankyrinG interacting proteins, such as voltage gated sodium channels to diffuse along the axon. So therefore, this disorganized distribution of AIS proteins can be due to the lack of specific microtubules domains, which modify the directional transport of kinesin-1, and do not allow the concentration of proteins at the AIS.

In conclusion, the distribution of HDAC6 in the distal region of the axon and its absence from the proximal region of the axon may regulate the formation of different microtubules domains in the axon. HDAC6 regulated activity at the distal axon can promote axonal growth, while microtubules at the proximal region of the axon can be more acetylated and allow the maintenance of the axon initial segment, necessary for polarized axonal traffic, tethering of AIS proteins and generation of neuronal action potentials. Further experiments will be necessary to understand how HDAC6 deacetylase activity is regulated at the axon.

## Supporting Information

Figure S1 HDAC6 inhibition and suppression increases tubulin acetylation in cultured hippocampal neurons. (A) 4 DIV hippocampal neurons treated with DMSO (control), TSA 100 nM or tubacin 10 µM, and stained for acetylated-α-tubulin. Note an increased gradient towards the growth cone in DMSO neurons that is not observed in TSA or tubacin treated neurons. Scale bar = 100 µm. (B) α-tubulin acetylation levels in cellular extracts of control neurons and neurons treated with TSA for 24 hours. (C) Fluorescence intensity of acetylated-α-tubulin along the axons of neurons treated as indicated in A. Graph represents the mean ± S.E.M. of acetylated-a-tubulin fluorescence intensity obtained from 5 neurons in each experimental condition. Each point represents the added fluorescence intensity of every 20 µm. (D) 4 DIV hippocampal neurons nucleofected with GFP plasmids expressing scramble or HDAC6 interference RNA. Neurons were stained with acetylated-α-tubulin (red). Scale bar  = 100 µm. (E) Fluorescence intensity of acetylated-α-tubulin along the axon in neurons nucleofected with scrambled or HDAC6 interference RNA 1 or 2. Fluorescence intensity of acetylated-α-tubulin along the axons of neurons treated as indicated in A. Graph represents the mean ± S.E.M. of acetylated-α-tubulin fluorescence intensity obtained from 5 neurons in each experimental condition. Each point represents the added fluorescence intensity of every 20 µm.
(2.10 MB TIF)

Figure S2 Quantification of HDAC6 expression levels in scramble shRNA and HDAC6 shRNA nucleofected neurons. (A) HDAC6 expression and DIC images of scramble shRNA and HDAC6 shRNA nucleofected neurons. Arrows indicate the soma of nucleofected neuron and arrowheads indicate the axon. Scale bar  = 50 µm. (B) Fluorescence intensity of HDAC6 along the axon in the neurons shown in A. (C) Normalized HDAC6 expression in axons of 50 neurons nucleofected with scramble shRNA or HDAC6 shRNA 1. ***p<0.001, paired t-test.
(3.23 MB TIF)

Figure S3 Non-active HDAC6-GFP reduces axonal growth. (A) 3 DIV hippocampal neurons nucleofected with HDAC6-GFP or non-active HDAC6-GFP plasmids and stained for tau-1. Scale bar  = 100 µm. (B) Mean axon length of the neurons shown in B, nucleofected with GFP, HDAC6-GFP or non-active HDAC6-GFP. Data represent the mean ± SEM of 3 independent experiments (100 GFP positive neurons/experimental condition and experiment). **p<0.01, paired t-test. (C) 4 DIV hippocampal neurons nucleofected with HDAC6-GFP or non-active HDAC6-GFP. Neurons were stained with anti-HDAC6 antibody (red). Note the colocalization of exogenous expressed HDAC6-GFP (green) and endogenous HDAC6 (red). Scale bar  = 100 µm.
(1.88 MB TIF)

Figure S4 HDAC6 impaired function interfere with N2a cells differentiation. (A) N2a cells transfected with pGFP-V-RS or pGFP-V-RS-shHDAC6 and differentiated for 2 days with 1 mM dibutyryl-cAMP. Transfected cells were identified by the GFP fluorescence and their morphology was determined staining cells for tyrosinated-α-tubulin. (B) Quantification of the percentage of GFP positive differentiated N2a cells transfected with the pGFP-V-RS or pGFP-V-RS-shHDAC6 plasmids. Data represent the mean and SEM of 3 independent experiments (200 GFP positive cells/experimental condition and experiment). *p<0.05, paired t-test. (C) N2a cells transfected with HDAC6-GFP or mutant non-active HDAC6-GFP and differentiated for 2 days with 1 mM dibutyryl-cAMP. Transfected cells were identified by the GFP fluorescence and their morphology was determined staining cells for tyrosinated-α-tubulin. (D) Percentage of GFP positive differentiated N2a cells transfected with the HDAC6-GFP or non-active HDAC6-GFP plasmids. Data represent the mean and SEM of 3 independent experiments (200 GFP positive cells/experimental condition and experiment). **p<0.01, t-test.
(1.87 MB TIF)
