# Supplementary figures and images for: Impaired Function of HDAC6 Slows Down Axonal Growth and Interferes with Axon Initial Segment Development
Source: PLoS One. 2010 Sep 23;5(9):e12908. doi: 10.1371/journal.pone.0012908 (PMC2944822; doi:10.1371/journal.pone.0012908)

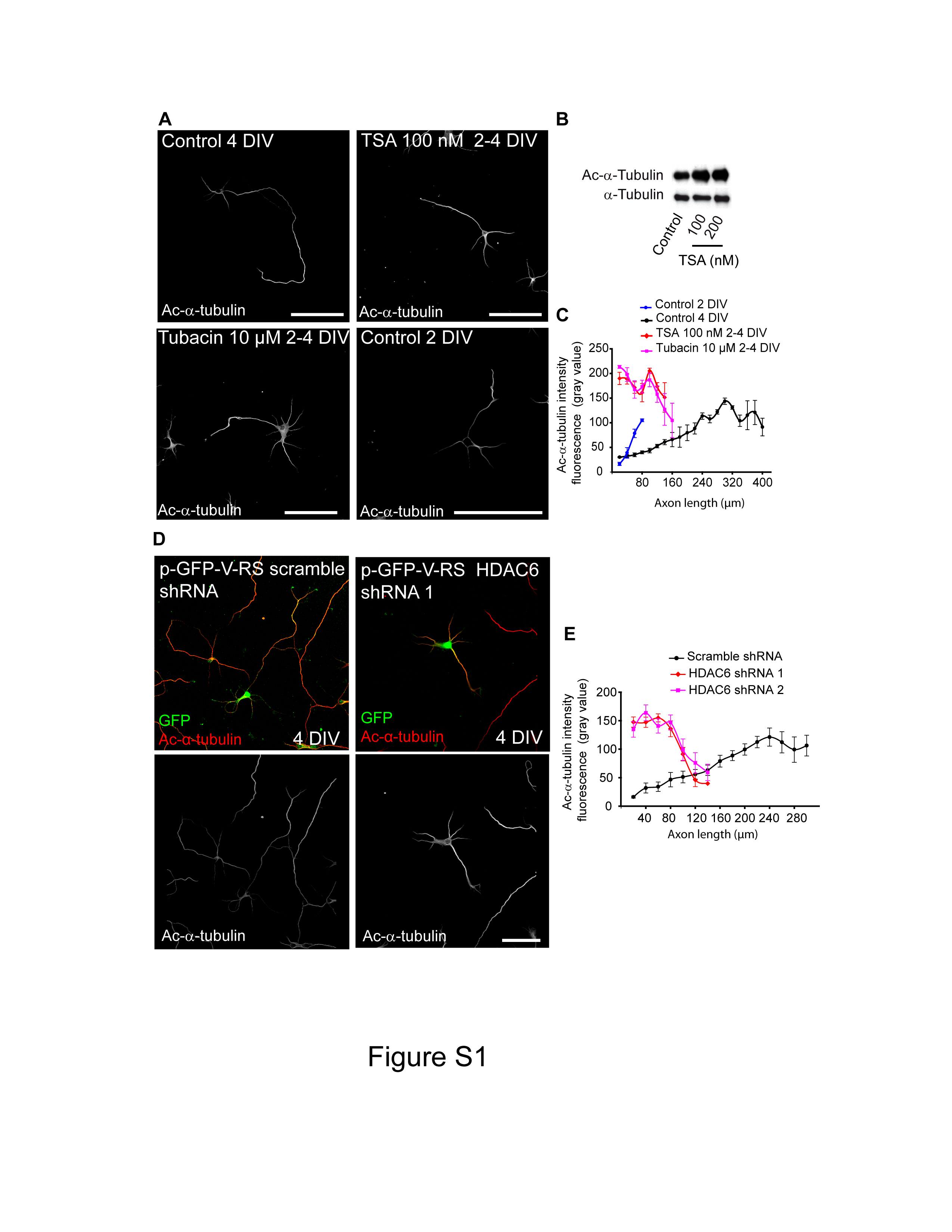

Supplement: Figure S1 — HDAC6 inhibition and suppression increases tubulin acetylation in cultured hippocampal neurons. (A) 4 DIV hippocampal neurons treated with DMSO (control), TSA 100 nM or tubacin 10 µM, and stained for acetylated-α-tubulin. Note an increased gradient towards the growth cone in DMSO neurons that is not observed in TSA or tubacin treated neurons. Scale bar = 100 µm. (B) α-tubulin acetylation levels in cellular extracts of control neurons and neurons treated with TSA for 24 hours. (C) Fluorescence intensity of acetylated-α-tubulin along the axons of neurons treated as indicated in A. Graph represents the mean ± S.E.M. of acetylated-a-tubulin fluorescence intensity obtained from 5 neurons in each experimental condition. Each point represents the added fluorescence intensity of every 20 µm. (D) 4 DIV hippocampal neurons nucleofected with GFP plasmids expressing scramble or HDAC6 interference RNA. Neurons were stained with acetylated-α-tubulin (red). Scale bar = 100 µm. (E) Fluorescence intensity of acetylated-α-tubulin along the axon in neurons nucleofected with scrambled or HDAC6 interference RNA 1 or 2. Fluorescence intensity of acetylated-α-tubulin along the axons of neurons treated as indicated in A. Graph represents the mean ± S.E.M. of acetylated-α-tubulin fluorescence intensity obtained from 5 neurons in each experimental condition. Each point represents the added fluorescence intensity of every 20 µm. (2.10 MB TIF) [file pone.0012908.s001.tif]

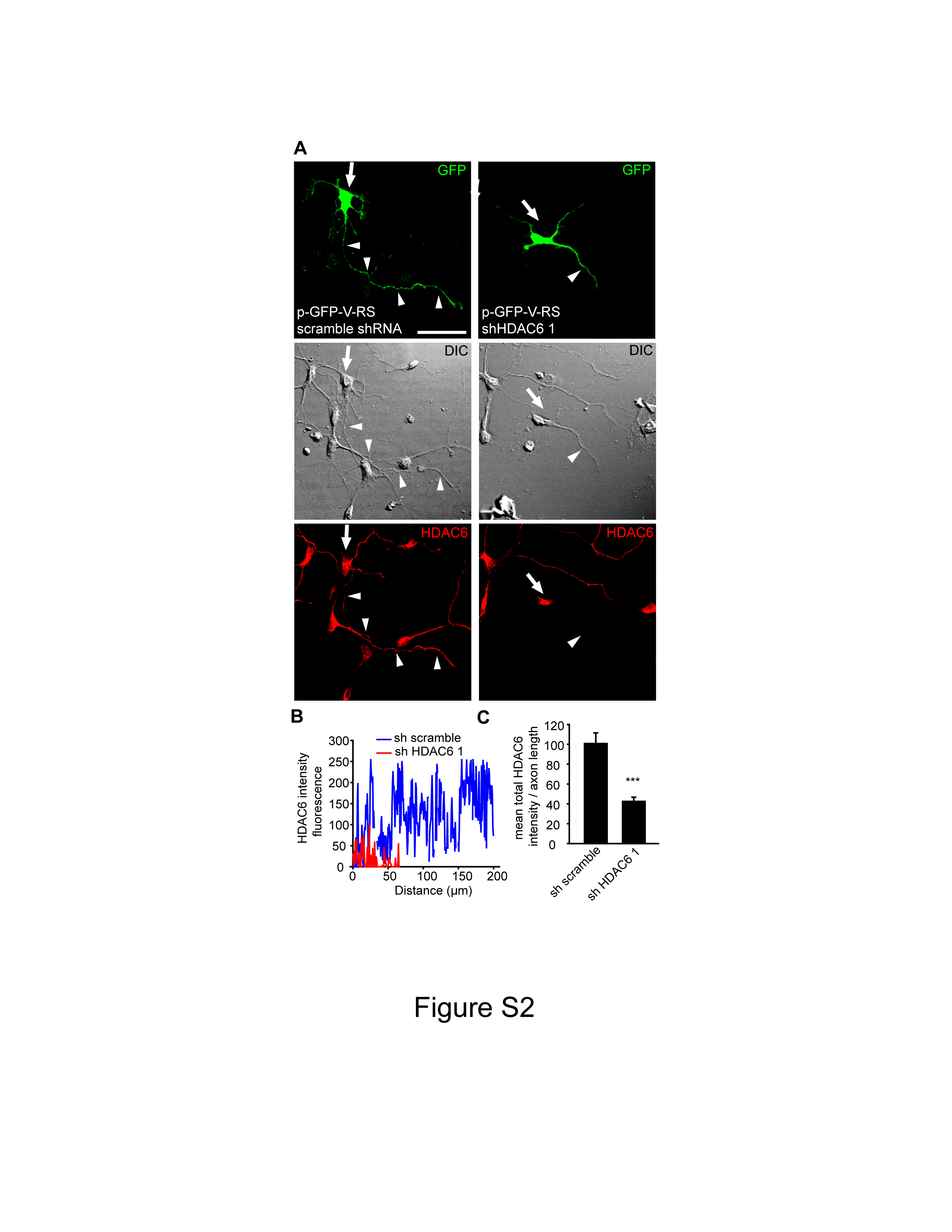

Supplement: Figure S2 — Quantification of HDAC6 expression levels in scramble shRNA and HDAC6 shRNA nucleofected neurons. (A) HDAC6 expression and DIC images of scramble shRNA and HDAC6 shRNA nucleofected neurons. Arrows indicate the soma of nucleofected neuron and arrowheads indicate the axon. Scale bar = 50 µm. (B) Fluorescence intensity of HDAC6 along the axon in the neurons shown in A. (C) Normalized HDAC6 expression in axons of 50 neurons nucleofected with scramble shRNA or HDAC6 shRNA 1. ***p<0.001, paired t-test. (3.23 MB TIF) [file pone.0012908.s002.tif]

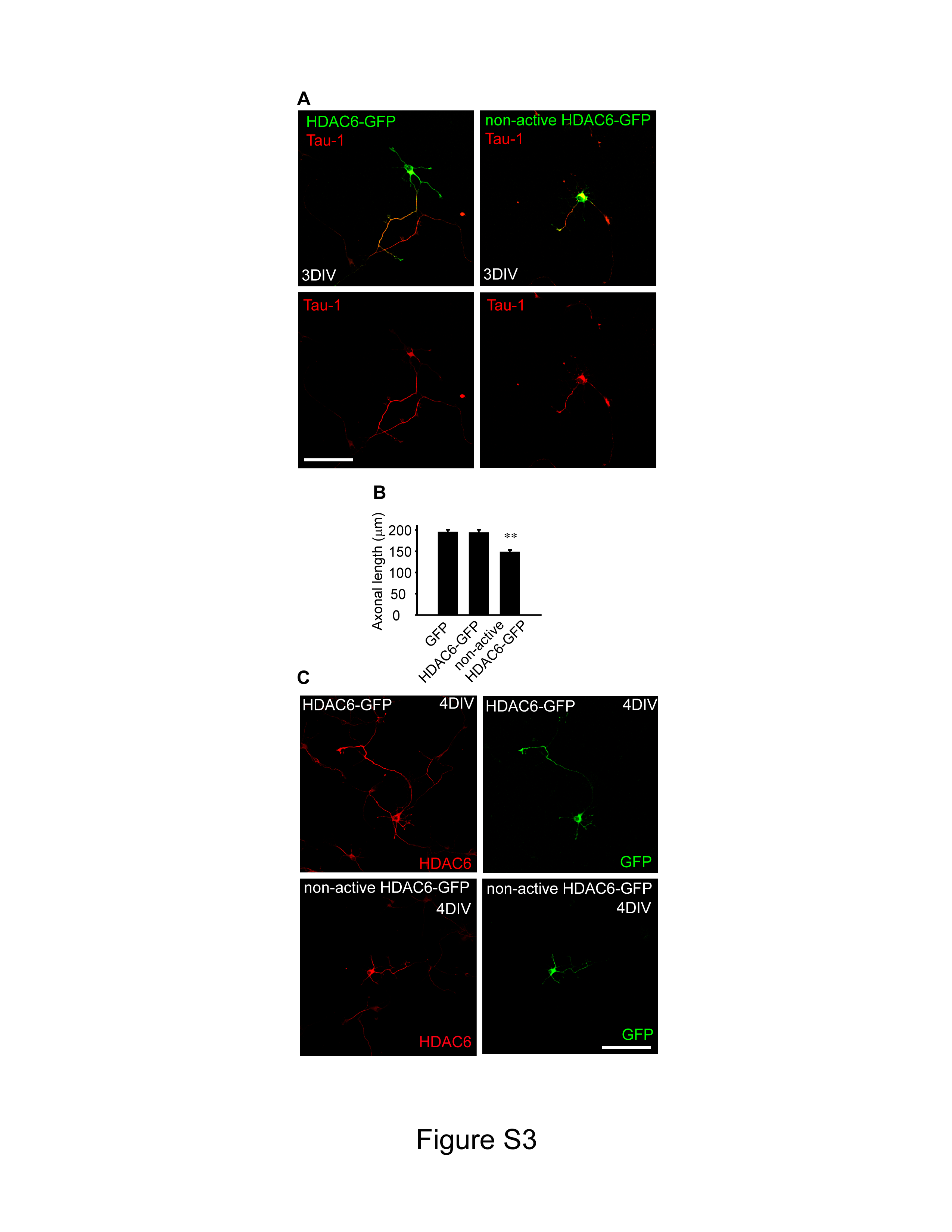

Supplement: Figure S3 — Non-active HDAC6-GFP reduces axonal growth. (A) 3 DIV hippocampal neurons nucleofected with HDAC6-GFP or non-active HDAC6-GFP plasmids and stained for tau-1. Scale bar = 100 µm. (B) Mean axon length of the neurons shown in B, nucleofected with GFP, HDAC6-GFP or non-active HDAC6-GFP. Data represent the mean ± SEM of 3 independent experiments (100 GFP positive neurons/experimental condition and experiment). **p<0.01, paired t-test. (C) 4 DIV hippocampal neurons nucleofected with HDAC6-GFP or non-active HDAC6-GFP. Neurons were stained with anti-HDAC6 antibody (red). Note the colocalization of exogenous expressed HDAC6-GFP (green) and endogenous HDAC6 (red). Scale bar = 100 µm. (1.88 MB TIF) [file pone.0012908.s003.tif]

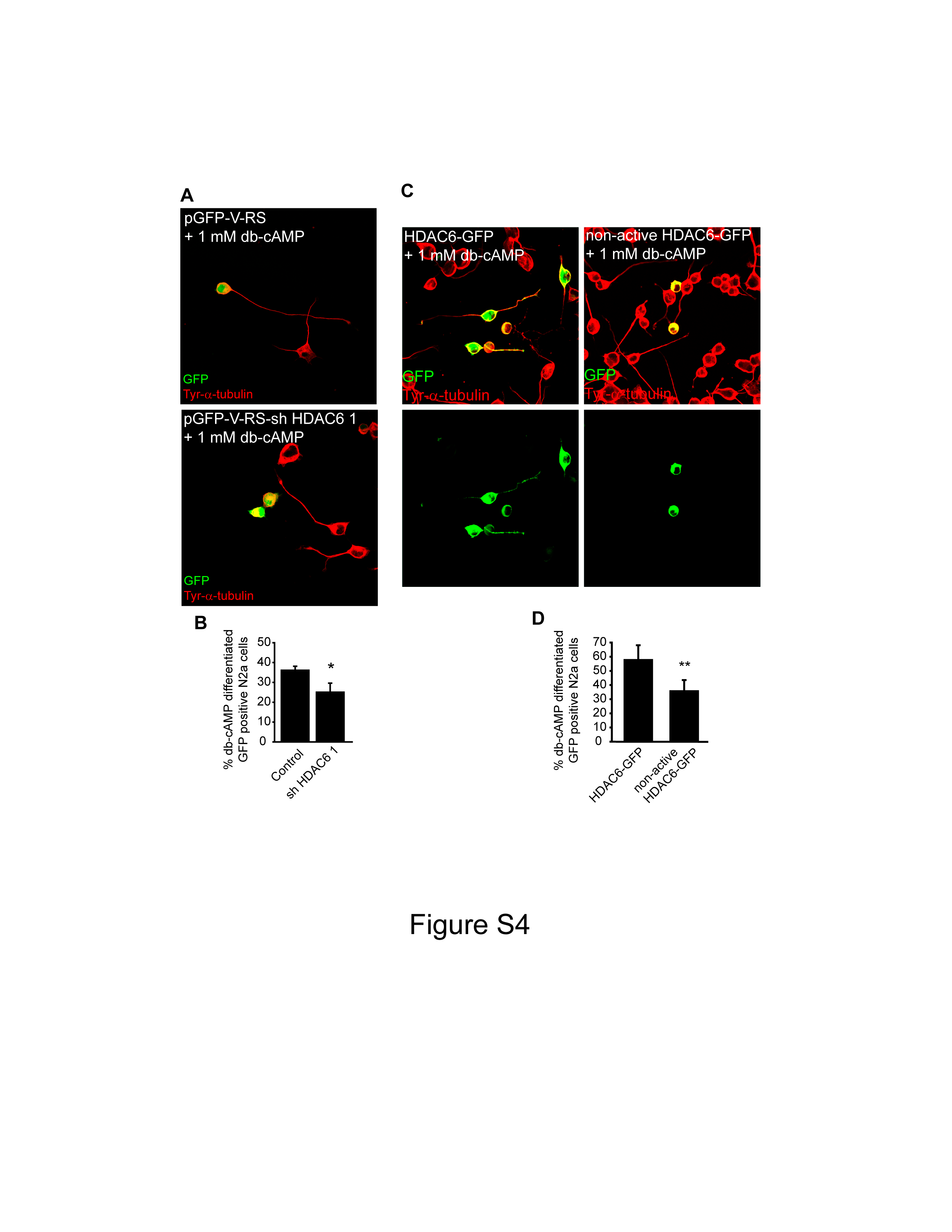

Supplement: Figure S4 — HDAC6 impaired function interfere with N2a cells differentiation. (A) N2a cells transfected with pGFP-V-RS or pGFP-V-RS-shHDAC6 and differentiated for 2 days with 1 mM dibutyryl-cAMP. Transfected cells were identified by the GFP fluorescence and their morphology was determined staining cells for tyrosinated-α-tubulin. (B) Quantification of the percentage of GFP positive differentiated N2a cells transfected with the pGFP-V-RS or pGFP-V-RS-shHDAC6 plasmids. Data represent the mean and SEM of 3 independent experiments (200 GFP positive cells/experimental condition and experiment). *p<0.05, paired t-test. (C) N2a cells transfected with HDAC6-GFP or mutant non-active HDAC6-GFP and differentiated for 2 days with 1 mM dibutyryl-cAMP. Transfected cells were identified by the GFP fluorescence and their morphology was determined staining cells for tyrosinated-α-tubulin. (D) Percentage of GFP positive differentiated N2a cells transfected with the HDAC6-GFP or non-active HDAC6-GFP plasmids. Data represent the mean and SEM of 3 independent experiments (200 GFP positive cells/experimental condition and experiment). **p<0.01, t-test. (1.87 MB TIF) [file pone.0012908.s004.tif]
